# Chronic UVB-irradiation actuates perpetuated dermal matrix remodeling in female mice: Protective role of estrogen

**DOI:** 10.1038/srep30482

**Published:** 2016-07-27

**Authors:** Katharina Röck, Simon Andreas Joosse, Julia Müller, Nina Heinisch, Nicola Fuchs, Michael Meusch, Petra Zipper, Julia Reifenberger, Klaus Pantel, Jens Walter Fischer

**Affiliations:** 1 Institut für Pharmakologie und Klinische Pharmakologie, Universitätsklinikum Düsseldorf, Heinrich-Heine-Universität Düsseldorf, Moorenstraße 5, 40225 Düsseldorf, Germany; 2 Department of Tumor Biology, University Medical Center Hamburg-Eppendorf, Martinistr. 52, 20246 Hamburg, Germany; 3 Department of Dermatology, Universitätsklinikum Düsseldorf, Heinrich-Heine-Universität Düsseldorf, Moorenstraße 5, 40225 Düsseldorf, Germany

## Abstract

Chronic UVB-exposure and declined estradiol production after menopause represent important factors leading to extrinsic and intrinsic aging, respectively. Remodeling of the extracellular matrix (ECM) plays a crucial role in both responses. Whether the dermal ECM is able to recover after cessation of UVB-irradiation in dependence of estradiol is not known, however of relevance when regarding possible treatment options. Therefore, the endogenous sex hormone production was depleted by ovariectomy in female mice. Half of the mice received estradiol substitution. Mice were UVB-irradiated for 20 weeks and afterwards kept for 10 weeks without irradiation. The collagen-, hyaluronan- and proteoglycan- (versican, biglycan, lumican) matrix, collagen cleavage products and functional skin parameters were analyzed. The intrinsic aging process was characterized by increased collagen fragmentation and accumulation of biglycan. Chronic UVB-irradiation additionally augmented the lumican, versican and hyaluronan content of the dermis. In the absence of further UVB-irradiation the degradation of collagen and accumulation of biglycan in the extrinsically aged group was perpetuated in an excessive matter. Whereas estradiol increased the proteoglycan content, it reversed the effects of the perpetuated extrinsic response on collagen degradation. Suspension of the intrinsic pathway might therefore be sufficient to antagonize UVB-evoked long-term damage to the dermal ECM.

Factors leading to dermal skin aging can be categorized into intrinsic and extrinsic responses. Intrinsic aging is triggered by physiological changes, like reduced estrogen (E_2_) levels over time at genetically determined rates^1^. Skin aging is accelerated after menopause, however upon E_2_ treatment positive effects like increased moisture, decreased wrinkling and improved wound healing become obvious in postmenopausal women^2^,^3^,^4^. Photoaging of the skin is mainly characterized by extrinsic responses inevitably occurring at sun exposed areas such as face, neck and hands. Even though only 5% of UVB light reaches the upper dermis, UVB has been shown to be a key factor of extrinsic aging by e.g. affecting dermal fibroblasts and their microenvironment^5^. However, up to date investigations interrogating the question whether the skin is able to recover from chronic UVB-irradiation are still lacking. Furthermore, it is unknown whether or not the processes of intrinsic and extrinsic aging are interconnected and if activation of the intrinsic pathway can abrogate remodeling processes induced by extrinsic aging.

One hallmark of skin aging is the altered composition of the extracellular matrix (ECM). Even though actinic cellular responses likely represent a consequence of complex matrix alterations, studies have focused mainly on single matrix components. Especially changes of the collagen matrix have been subject of intensive studies throughout the last years. It is known that UVB-light induces the proteolytic cleavage of collagen fibrils^6^. Repeating damage through successive UV-irradiations is only repaired insufficiently as a result of glycylation products which cannot be degraded^7^. Both accumulation of these micro-lesions and additional loss of total skin collagen with age essentially contribute to the functional impairments of the aged collagen matrix, resulting in skin laxity and wrinkles^8^,^9^. Apart from collagen, UVB has been reported to affect other dermal ECM components, in particular hyaluronan (HA) and proteoglycans (PG)^10^,^11^. HA is an unbranched polymeric carbohydrate consisting of alternating disaccharide units (D-glucuronic acid beta(1–3)-D-N-acetyl-glucosamine beta(1–4)). Through interaction with its receptors (e.g. CD44) and its binding partners such as tumor necrosis factor stimulated gene 6, HA is thought to critically determine cellular phenotypes^12^,^13^,^14^. Histological and biochemical studies of the HA metabolism in the course of actinic aging reported contradictory results depending on the intensity, duration and amount of irradiations. It was shown that acute UVB-irradiation leads to an increase of dermal HA^15^ whereas studies investigating chronic irradiation reported a decrease of dermal HA^16^,^17^.

Versican (VCAN), a large chondroitin sulphate proteoglycan is a known binding partner of hyaluronan. Multiple VCAN molecules are able to bind to one HA chain whereby large networks are formed, which are e.g. known to regulate the proliferative and migratory phenotype of mesenchymal stem cells^18^. It has recently been demonstrated that VCAN accumulates after UVB-irradiation^19^. In response to acute UV-irradiation VCAN appears to be essential for the establishment of a micromilieu, which facilitates repair- and remodeling processes to support wound healing^20^.

Regarding actinic skin aging little is known about the function and regulation of the small leucine rich proteoglycans (SLRP) biglycan (BGN) and lumican (LUM). Both SLPRs have been shown to bind collagen and are essential for the collagen fibril formation^21^. Mice deficient of the respective proteins reveal a skin phenotype resembling the Ehlers-Danlos Syndrome^22^. In addition, LUM is a known regulator of cellular proliferation and migration^22^. Apart from its binding properties to collagen, BGN stimulates multifunctional pro inflammatory immune responses^23^. In the corneal epithelium of rabbits an accumulation of BGN was found in response to UV-exposition^24^.

Information about changes of the ECM due to loss of E_2_ in the course of intrinsic aging are up to date very limited. E_2_ has been shown to antagonize the loss of dermal collagen in ovariectomized mice^25^,^26^ and topical application of E_2_ induces pro-collagen synthesis in a TGF-ß mediated manner in aged human skin^27^. Furthermore, E_2_ induces the release of epidermal growth factor thereby stimulating VCAN and HA expression in the dermis of UVB-irradiated skin^28^.

Aim of the present study was to determine whether remodeling of the ECM composition is an ongoing process even after abrogation of chronic UVB or if desistance from UVB results in a long term recovery of the dermal ECM. Furthermore the effects of E_2_ on the complex changes of the dermal ECM in response to chronic UVB-irradiation was analyzed using ovariectomized and E_2_ treated hairless mice.

## Results

### Sustained loss of collagen during UVB-free recovery

**Figure 1:**
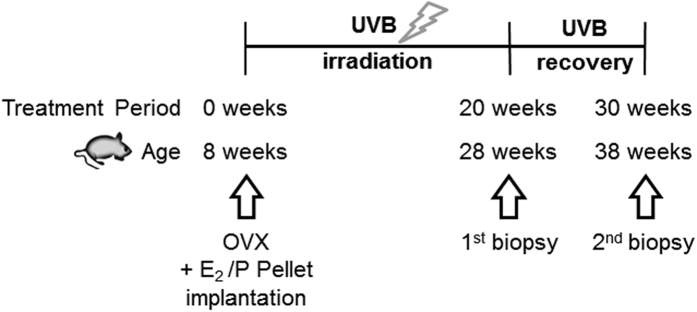
Experimental design. Hairless skh-1 mice were ovariectomized (OVX; X) at the age of 8 weeks (wk). OVX mice received either a subcutaneous placebo pellet (P) as control or a subcutaneous slow release pellet (1.1 μg E_2_/day/mouse) (E_2_). Next, mice were subjected to UVB-irradiation (three times 1 minimal erythema dose, weekly) for 20 weeks. At the age of 28 weeks, skin biopsies were obtained and analyzed. A second biopsy was taken further 10 weeks after the last irradiation at the age of 38 weeks in order to evaluate long term recovery from chronic irradiation.

Two specific questions were addressed for the first time: (i) is the UVB-induced degeneration of the ECM reversible during a 10 week recovery period and (ii) does E_2_ as intrinsic factor mediate specific effects on the ECM during UVB-irradiation and during the UV-free recovery period. To address these questions mice were irradiated with UVB three times a week (1 minimal erythema dose (80 mJ/cm^2^) for 20 weeks and the UVB-mediated effects on the matrix were determined. Subsequent to the 20 weeks of irradiation mice were allowed to recover 10 weeks without UVB-irradiation and analyzed with respect to changes of the dermal matrix afterwards (Fig. 1). In parallel half of the mice received E_2_ substitution to elucidate the effects of E_2_ on acute UVB-mediated changes and during the recovery period. Age matched mice plus minus E_2_ substitution without UVB-irradiation served as controls.

**Figure 2:**
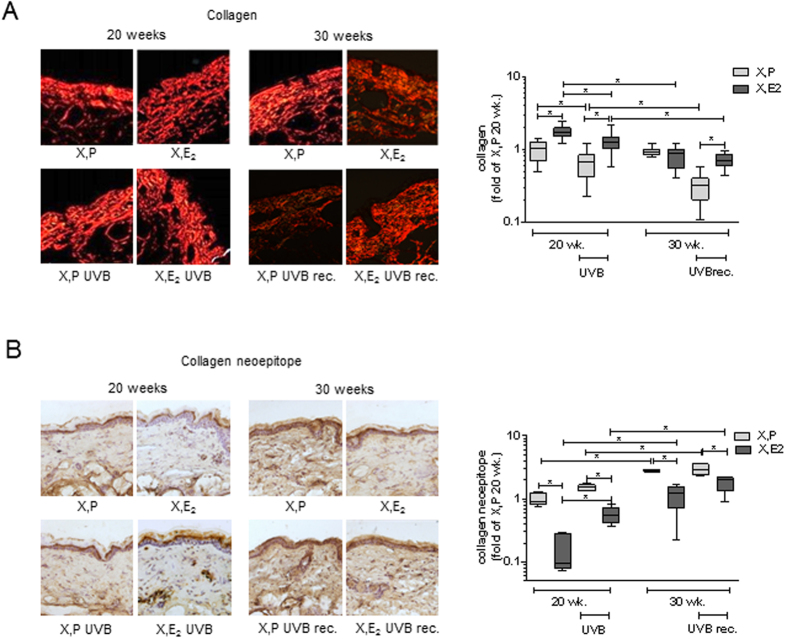
Up regulation of collagen and reduction of collagen fragments in response to E_2_ *in vivo*. Collagen expression was determined in skin biopsies from hairless skh-1 mice that were treated as detailed in Fig. 1. After 20 and 30 weeks (wk), skin biopsies were obtained, and the amount of collagen was analyzed. (**A**) Picosiriusred staining for collagen and quantification in the papillary dermis. (**B**) Immunohistochemical staining of collagen neoepitopes and quantification in the papillary dermis. 200x magnification; n = 7–12, *p < 0.05 as indicated.

Interestingly in the intrinsically aged group 20 weeks of E_2_ treatment significantly elevated dermal collagen in comparison to ovariectomized mice. However, whereas the collagen content of the dermis was maintained in the X, P group it was reduced to X, P level after 30 weeks in the estradiol treated animals. Chronic UVB-irradiation caused a reduction of the dermal collagen content by ~35% in the murine dermis in both X, P and X, E_2_ treated mice compared to the respective control. Subsequently it was investigated whether the dermal collagen matrix was able to recover from chronic UVB-irradiation by a UVB free period of additional ten weeks or if the remodeling process is ongoing. Unexpectedly the collagen content further declined ~70% compared to X, P 20 wk. In comparison the additional loss of collagen during the recovery period was not as distinct in the X, E_2_ UVB recovery animals, where the collagen content matches the intrinsically aged control after 30 weeks (Fig. 2A). Collagen type I mRNA levels were not significantly altered by either UVB or E_2_ treatment (supplement 1).

Loss of collagen matrix can either result from reduced collagen *de novo* synthesis or from proteolytic cleavage by collagenases like matrix metalloproteinases (MMP). Both mechanisms are in place during intrinsic and extrinsic aging. It has been shown previously that MMPs are increased during photo aging^29^. E_2_ on the other hand has been associated with a reduction of proteolytic activity of MMPs in osteoarthritis^30^. Therefore we analyzed the accumulation of collagen neoepitopes as indicator of increased collagen fragmentation.

In concordance with the E_2_ induced increase of dermal collagen in the intrinsically aged group (Fig. 2A) the content of collagen neoepitopes was found to be altered reciprocally to total collagen after 20 weeks (Fig. 2B). After 30 weeks the amount of collagen neoepitopes was still increased in the X, P group compared to X, E_2_ however did not match the total collagen content as seen in Fig. 2A. Collagen neoepitopes were slightly increased by 20 weeks of UVB-irradiation in both groups and even more important continued to increase throughout the UVB-free recovery period. The collagen degrading enzymes MMP 3, 9 and 13 (functional replacement of MMP1 in mouse pathobiology ) have been described to be affected by UV-irradiation^31^. Therefore their expression patterns were analyzed by qRT-PCR. Whereas MMP 3 and 9 were not altered, MMP13 was reduced by E_2_ treatment (supplement 1). The loss of collagen in response to UVB-irradiation cannot be explained by regulation of the MMP 3, 9 or 13 mRNA expression at the indicated time points.

Taken together, collagen fragmentation is increased over time in both the intrinsically and extrinsically aged murine dermis. Of note, collagen fragmentation continues in an excessive manner after desistance from UVB. E_2_ suppressed collagen fragmentation during both aging processes.

### Hyaluronan is not altered by chronic UVB irradiation however is sensitive to E_2_

**Figure 3:**
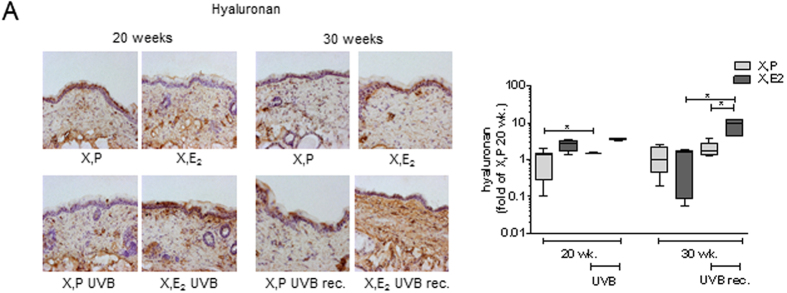
Induction of HA by E_2_ *in vivo*. HA expression was determined in skin biopsies from hairless skh-1 mice that were treated as detailed in Fig. 1. After 20 and 30 weeks (wk), skin biopsies were obtained, and the amount of HA was analyzed. (**A**) Affinity histochemistry for HA and quantification in the papillary dermis. 200x magnification; n = 7–12, *p < 0.05 as indicated.

Subsequently the regulation of dermal HA was characterized. After 20 and 30 weeks E_2_ increased the amount of HA in the intrinsically aged dermis compared to X, P. No alterations of the HA matrix could be detected between 20 and 30 weeks within the respective groups (X, P 20 wk. vs 30 wk., XE_2_ 20 wk. vs 30 wk). Furthermore, 20 weeks of UVB irradiation did not change the HA content of the murine dermis. Interestingly 10 weeks after the last irradiation HA was increased in an excessive manner in the E_2_ group compared to X, P UVB (Fig. 3).

### E_2_ and UVB up-regulate proteoglycan content in dermal matrix

**Figure 4:**
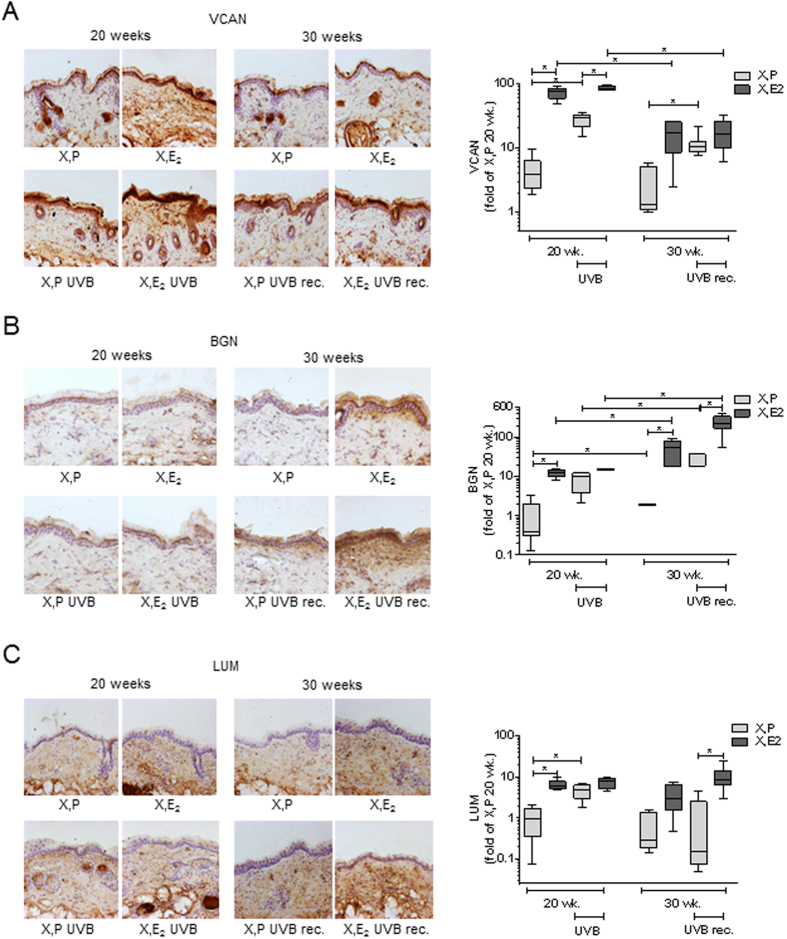
Differential regulation of proteoglycans by E_2_ und UVB *in vivo*. Versican (VCAN), Biglycan (BGN) and Lumican (LUM) expression was determined in skin biopsies from hairless skh-1 mice that were treated as detailed in Fig. 1. After 20 and 30 weeks (wk), skin biopsies were obtained, and the proteoglycan content was analyzed. (**A**) Immunohistochemistry for VCAN and quantification in the papillary dermis. (**B**) Immunohistochemistry for BGN and quantification in the papillary dermis. (**C**) Immunohistochemistry for LUM and quantification in the papillary dermis. 200x magnification; n = 7–12, *p < 0.05 as indicated.

Next, the regulation of dermal proteoglycans VCAN, BGN and LUM was analyzed. VCAN is a HA-binding proteoglycan that forms large hydrated ECM networks. Immunostaining of VCAN revealed that E_2_ substitution caused an intensive VCAN accumulation in non-irradiated skin after 20 and 30 weeks of E_2_ treatment. The VCAN content did not change between 20 and 30 weeks in the X, P group however was reduced at the 30 week time point when comparing X, E_2_ 20 wk. with X, E_2_ 30 wk. (Fig. 4A). Furthermore VCAN was found to decline during the UVB-free recovery period in both X, P and X, E_2_ treated mice. Apart from the dermal compartment VCAN accumulation was also observed in the epidermis in an E_2_ and UVB responsive manner. The increase of VCAN after E_2_ treatment was also reflected by the mRNA expression profile (supplement 1).

BGN and LUM belong to the family of small leucine rich proteoglycans which are known binding partners of collagen. BGN was shown to be strongly responsive to E_2_ as it was significantly increased after 20 and 30 weeks of E_2_ treatment (Fig. 4B). No change could be detected between 20 and 30 weeks in the ovariectomized groups, however BGN was severely increased in the E_2_ treated intrinsically aged group (20 weeks vs 30 weeks). Interestingly BGN increased as a result of UVB-irradiation and continued to increase during the UVB-free recovery period.

Compared to X, P dermal LUM was elevated by E_2_ after 20 and 30 weeks (Fig. 4C). LUM was not altered in between 20 and 30 weeks in the X, P animals. In X, P and X, E_2_ treated animals LUM increased in response to 20 weeks of UVB and stayed elevated even during recovery. Similar regulation patterns as seen in the immunohistochemical stainings were detected when analyzing the mRNA expression patterns of both BGN and LUM (supplement 1).

### Differential effects of proliferation and inflammation during the UVB-free recovery phase

**Figure 5:**
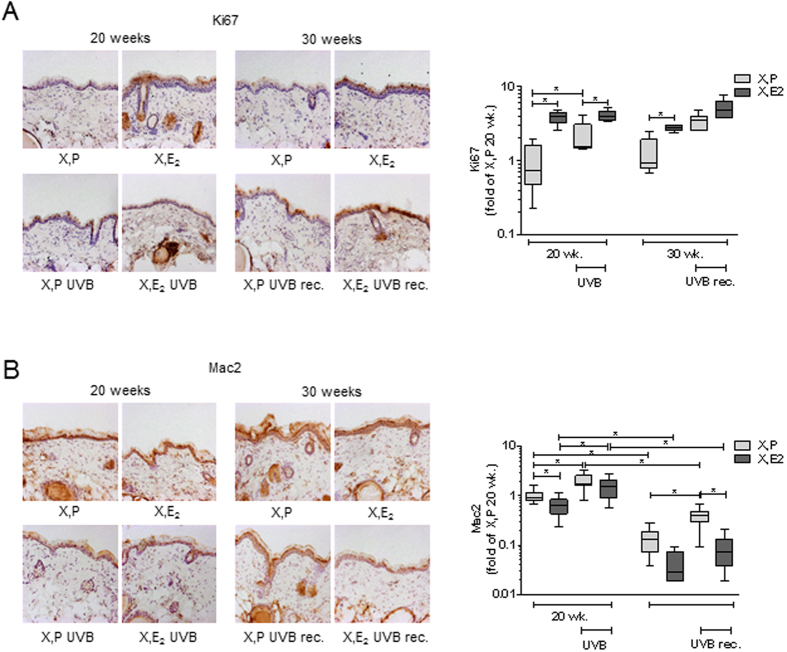
Proliferatory and inflammatory response to E_2_ and UVB *in vivo*. The proliferation and inflammatory response was analyzed in skin biopsies from hairless skh-1 mice that were treated as detailed in Fig. 1. After 20 and 30 weeks (wk), skin biopsies were obtained. (**A**) Immunohistochemistry for proliferation marker Ki67 and quantification in the papillary dermis. (**B**) Immunohistochemistry for Mac-2 and quantification in the papillary dermis. 200x magnification; n = 7–12, *p < 0.05 as indicated.

Next it was attempted to associate the quantitative and qualitative remodeling of the dermal matrix with changes in proliferation and inflammation. For this purpose the proliferation of dermal fibroblasts was determined by Ki67 immunostaining (Fig. 5A) in the dermal compartment. E_2_ elevated the amount of Ki67 positive cells after 20 and 30 weeks of treatment. In between the 20 and 30 week time point proliferation was not altered. Interestingly, UVB-irradiation resulted in an increase of the Ki67 positive cells in the dermis of X, P animals which was not found in the X, E_2_ group. As a late sequel of irradiation proliferation was further increased in X, P mice as well as X, E_2_ mice.

The infiltration of MAC-2 positive immune cells in the dermis (Fig. 5B) was reduced by E_2_ treatment after 20 and 30 weeks. In addition MAC-2 staining was decreased over time (20 vs 30 weeks) in both X, P and X, E_2_ treated animals. As expected UVB-irradiation strongly induced MAC-2 staining. Of note, ten weeks after the last irradiation the amount of MAC-2 positive cells was almost decreased to the amount found in the non-irradiated control animals. These results suggest that the inflammatory response occurs acutely during the irradiation and declines during the UVB-free recovery phase.

All considered the matrix was characterized by increased content of collagen fragments and SLRPs (BGN, LUM) and by decreased collagen after UVB-irradiation and after UVB-recovery. This matrix remodeling was associated with increased dermal cell proliferation. VCAN and inflammation (as evidenced by MAC2) were the only parameter that appeared to dramatically recovered during the 10 weeks without UVB-irradiation, possibly hinting towards a role of VCAN in UVB-induced inflammation.

### Matrix remodeling is associated with changes of functional skin parameters

**Figure 6:**
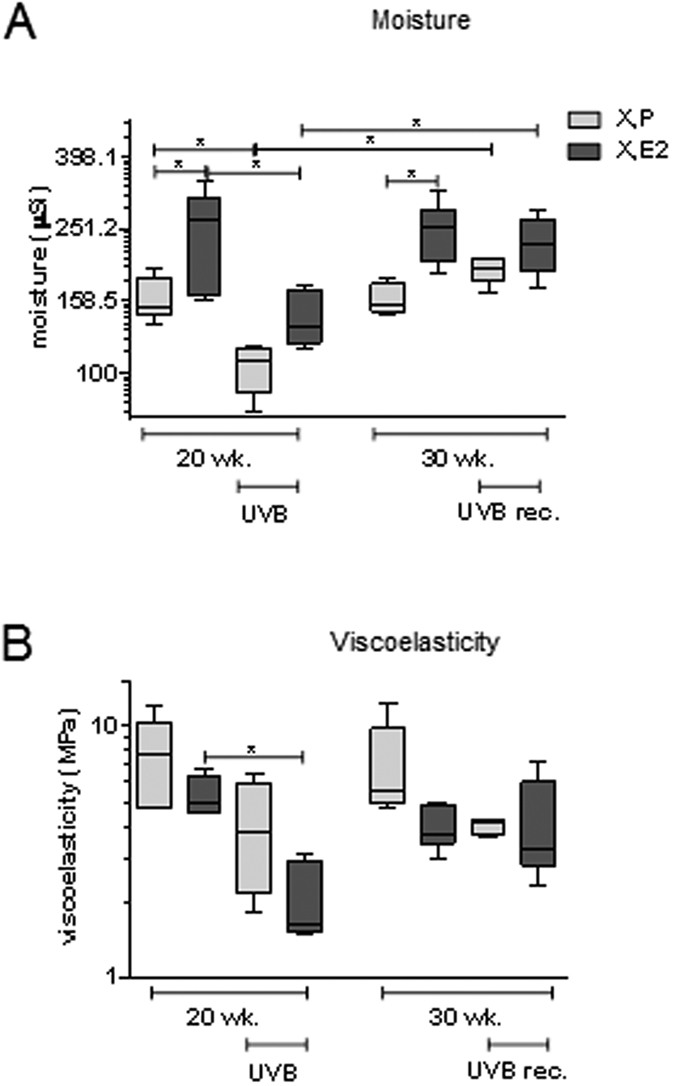
Functional skin parameters in response to E_2_ and UVB *in vivo*. After 20 and 30 weeks (wk) moisture and viscoelasticity was measured in skin from hairless skh-1 mice that were treated as detailed in Fig. 1. (**A**) Moisture content of the skin. (**B**) Viscoelasticity; n = 7–12, *p < 0.05 as indicated.

Furthermore it was attempted to link the specific changes in the composition of the extracellular matrix intrinsic and extrinsic aging to skin moisture and viscoelasticity (Ve). Moisture content of the skin was elevated by E_2_ but was not altered during in the intrinsically aged groups between 20 and 30 weeks (Fig. 6A). After 20 weeks of UVB-irradiation moisture was markedly reduced in both X, P and X, E_2_ treated animals, however again raised to control level at the end of the recovery period.

Ve was not significantly altered by E_2_ or over the course of additional 10 weeks. UVB-irradiation decreased Ve the X, E_2_ treated animals, however recovered to the same level as the intrinsically aged control during the UVB free period.

### Statistical analysis reveals distinct matrix alterations

**Figure 7:**
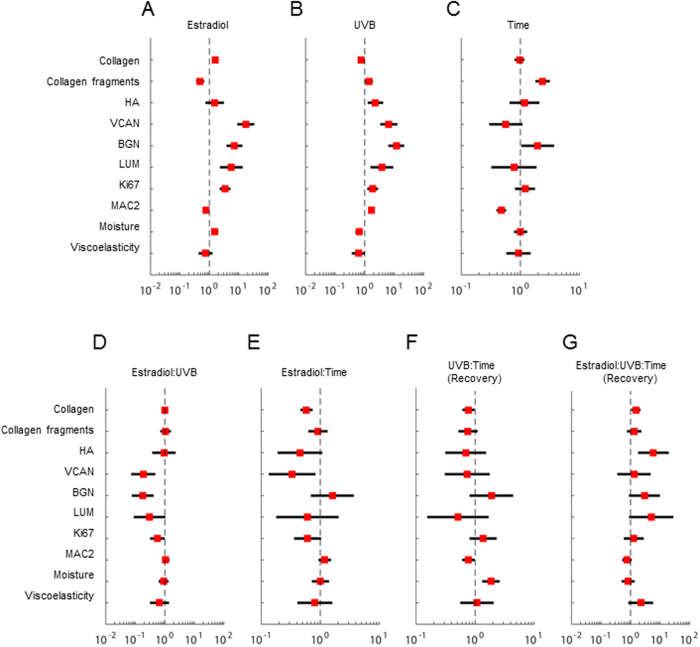
Statistical analysis of matrix associated aging processes. Odds ratios. Estradiol, UVB, and Time treatment effects on viscoelasticity, moisture, MAC2, Ki67, LUM, BGN, VCAN, HA, Collagen Fragment, and Collagen depicted as odds ratios (red) with 95% confidence intervals (black horizontal lines). Panel A–C: main effects of treatments on matrix components and functional skin parameters. Panel D–G: effects caused by interaction between treatments.

Finally a Generalized Linear Model (GLM) for 2 × 2 × 2 factorial treatment design was applied to comprehensively investigate the sole effects of E_2,_ UVB and time as well as their interactions. In summary, E_2_ was found to increase the dermal proteoglycans and collagen content possibly due to reduced collagen fragmentation. Intrinsic aging (time) was characterized by an increase of biglycan and collagen fragmentation. Additionally to the intrinsic effect UVB-treatment induced lumican and versican and reduced collagen. Interestingly E_2_ was found to reverse the effects of UVB and time when interacting with both parameters tending to even reduce matrix components which were unaffected or increased with regard to the single parameters (e.g. collagen Fig. 7E). Whereas most matrix components recovered during the UVB free period the effect on biglycan and collagen was perpetuated. Of note, the remodeling process inflicted on collagen was abrogated by E_2_ (Fig. 7G) whereas the effect on biglycan was tendentially further increased.

Taken together these findings reveal a dramatic qualitative and quantitative remodeling of the collagen and proteoglycan matrix during extrinsic aging that is partially sustained and even perpetuated in the absence of further UVB-irradiation. Suspension of the remodeling of the collagen matrix as well as further modulation of the BGN matrix by E_2_ might be sufficient to antagonize UVB-evoked long-term damage to the dermal ECM.

## Discussion

Various studies have provided information about structural alterations of the skin during the intrinsic and extrinsic aging process. One hallmark of dermal aging are changes of the extracellular matrix (ECM). Even though investigations up to date have mainly focused on single ECM molecules, it appears likely that pathophysiological and physiological aging processes involve changes of a network of various matrix molecules that will affect cellular responses. In addition, it is still unknown if the ECM composition is able to recover from chronic UVB-irradiation and whether estrogen as a major repressor of the intrinsic aging process is also able to modify these extrinsic responses.

Here we show for the first time that complex matrix remodeling processes induced by chronic UVB-irradiation are pursued even after a subsequent UVB-free period of ten weeks and E_2_ indeed is a modulator of these processes.

Our data suggest that after 20 weeks of irradiation collagen degradation is activated and stays activated even in the absence of UVB-irradiation. LUM core-protein has been reported to reduce collagen synthesis and inhibition of *de novo* collagen synthesis is abrogated in LUM deficient mice^32^.

It was reported recently that dimerized LUM molecules can bind to collagen fibrils and prevent cleavage by MMPs due to steric hindrance^33^. Additionally, BGN has been shown to be essential for the accurate formation of collagen fibers which is of importance for the maintenance of the integrity of the skin^34^. Of note, high amounts of LUM and BGN were accompanied by reduced amounts of collagen fragments.

Activated break down of collagen is most likely the cause for the sustained loss of collagen. Collagen degradation in the dermis is primarily contributed to matrix metalloproteinases (MMP) 1 (murine MMP 13), −3 and −9^35^. MMP levels are raised in response to UVB-irradiation and as a general phenomenon in the course of dermal aging^36^. In the past collagen fragments have been shown to repress *de novo* collagen synthesis and further induce MMP 1 and −3^37^. The processes might result in a cumulative, irreversible damage of the collagen structure which is characterized by further reduction of the collagen content even 10 weeks after the last UVB-irradiation. Interestingly, UVB-irradiation did not lead to an altered mRNA expression of MMP 3, −9 or −13 in the present study. However, this might be explainable by an earlier activation of MMPs or a biochemical activation independent of the mRNA level.

The protective effects of estrogen on the integrity of the skin, which is in particular obvious after the initiation of estrogen treatment of post-menopausal women has been described in various studies^38^. E_2_ increases skin thickness, augments moisture and reduces wrinkling^39^,^40^. E_2_ treatment has been shown to prevent loss of collagen and increased *de novo* collagen synthesis during actinic aging^26^. In line with our results E_2_ treatment might furthermore act by abolishing collagen degradation by reduction of MMP activity^40^,^41^.

In addition, loss of HA from UVB irradiated dermis appears to be part of the postmenopausal aging process^28^. The reduction of collagen degradation by E_2_ could be one possible mechanism behind the elevation of HA levels as seen after E_2_ treatment in all experimental groups. Additionally E_2_ is known to induce HA synthesis in a paracrine mechanism by inducing EGF release in epidermal keratinocytes^28^. Moreover, a mutual interaction between HA and E_2_ is conceivable. Hence, E_2_ does not solely induce HA-synthesis, but HA causes an increase of E_2_-receptor activation through CD44 and ERK2 phosphorylation^42^.

It has been demonstrated that the HA-binding proteoglycan VCAN which was found to be highly responsive to E_2_ and UVB-irradiation is able to directly activate CD44 thereby stabilizing the integrity of the HA-matrix^43^ and possibly facilitating its effect on E_2_-receptor activation. The responsiveness of VCN to both UVB and E_2_ is diminished at the 30 week time point possibly being involved in the late reduction of dermal HA content. Protection of the HA-matrix at the earlier time point by VCAN is plausible by the reduction of oxidative stress through VCAN overexpression^44^. HA degradation into HA fragments by reactive oxygen species might thereby be prevented^45^,^46^. Furthermore, HA is bound to VCAN through specific binding domains termed link modules, thus stabilizing the HA matrix.

Whereas UVB-induced the amount of Mac2 positive cells in the dermis, E_2_ acted as a suppressor of inflammation. Even though a strong reduction of the Mac2 signal intensity was detected at 30 weeks, the regulation pattern was found to be similar to the 20 week time point. It is conceivable that alterations of the matrix composition result in a modified invasion pattern of inflammatory cells. Furthermore, macrophages secrete inflammatory mediators and MMPs which can accelerate dermal aging. Exemplary LUM deficient mice reveal reduced invasion of macrophages and concentration of inflammatory cytokines^47^, whereas HA appears to be a key modulator of wound healing processes^48^,^49^. Both UVB and E_2_ induce the release of TGF-β and other cytokines in fibroblasts *in vitro* and *in vivo*^50^, which in turn upregulate HA, BGN, VER and collagen. All of those ECM components have been associated with a pro proliferative phenotype in fibroblasts^47^,^51^,^52^. Cytokine expression levels have not been subject to this study, however could explain the perpetuated effect of UVB and E_2_ treatment in a direct or ECM-dependent indirect manner.

As functional readout, skin moisture and viscoelasticity were measured. UVB-irradiation reduced skin moisture however the values recovered 10 weeks after the last UVB-irradiation to the 20 week control level. E_2_ elevated the moisture of the skin in all experimental settings. Possible cause for the effects on moisture is the corresponding regulation of dermal HA. In contrast E_2_ as well as UVB reduced the skin viscoelasticity. Importantly, the values did not return to the initial values during the UVB-pause. Higher stiffness of the skin as presented by low levels of Ve might evolve by the increase of PG content in the dermis after UVB or E_2_ treatment and thereby induction of cross linkage of different matrix molecules. The loss of skin tension of 80–90% has been reported upon a lack of SLRPS^53^.

UVB photons are about 1000 times more energetic than UVA photons and are responsible for sunburn, tanning and photocarcinogenesis after sun exposure. However, it needs to be emphasized that UVA is suspected to play a proportionately larger role in photoageing because of its greater abundance in sunlight^54^. Future studies exploring the influence of UVA on the ECM composition might contribute to the further understanding of the aging process.

In conclusion, this study clearly demonstrates that several actinic remodeling processes of chronic UVB-irradiation are maintained even 10 weeks following the last UVB-irradiation. E_2_ is a strong inductor of several matrix components and might help to suspend these ongoing remodeling processes.

## Materials and Methods

### UVB-irradiation of Mice

Female hairless Skh:Hr1 mice (Charles-River) were housed according to standard procedures at the “Zentrales Tierlabor der Universität Düsseldorf”. All animal experiments were approved by the local ethical committee (LANUV, Das Landesamt für Natur, Umwelt und Verbraucherschutz Nordrhein-Westfalen) for animal experiments (approval number 8.87-50.10.34.08.022). All methods were carried out in “accordance” with the approved guidelines.

Mice were bilaterally ovariectomized (OVX, X) at the age of 8 weeks as described previously^51^. Subcutaneous slow-release hormone pellets (Innovative Research of America) prepared to dispense 1.1 μg/d 17-β-estradiol (E_2_) were subcutaneously implanted to ensure a constant liberation of E_2_ for the duration of the experimental period. Placebo (P) pellets served as control. After OVX and pellet implantation half of the mice were irradiated with UVB light and the other half served as non–irradiated controls. Thus four experimental groups (Fig. 1A) were compared in total: 1) OVX, placebo (X, P), 2) OVX, E_2_ (X, E_2_), 3) OVX, placebo, UVB (X, P UVB) and 4) OVX, E_2_, UVB (X, E_2_ UVB).

UVB-exposure was performed in an irradiation chamber as described previously^52^ using UV lamps with fluorescent bulbs (280 to 320 nm with a peak at 313 nm TL 20W/12; Philips, Eindhoven, The Netherlands). UVB-irradiation was performed three times per week at a dose of 80 mJ/cm^2^ (irradiation time 1 minute 36 seconds) equaling 1 minimal erythema dose (MED) over a period of 20 weeks (Fig. 1) followed by a recovery phase of 10 weeks without irradiation. The light intensity was determined by means of a UV meter (Waldmann, Villingen-Schwennigen, Germany). A 1 × 1.5 cm^2^ sized skin biopsy from the dorsal back of all animals was obtained from control and UVB-irradiated animals at the age of 28 weeks (20 weeks of irradiation) and at 38 weeks (further 10 weeks of recovery from UVB). Anesthesia was performed using i.p. Xylazin/Ketamin injection. Euthanasia was performed by cervical dislocation.

### Histology

Skin biopsies were frozen in tissue freezing medium (Leica Nussloch, Bensheim, Germany) and 14 μm thick cryosections were prepared. Affinity histochemistry of HA was performed with bovine HA binding protein (bHABP, Seikagaku, Tokyo, Japan), detected with biotin-labeled streptavidin (2 μg/ml, Calbiochem, Bad Soden, Germany). For immunohistochemistry the following primary antibodies were used: versican (LF99, 1:400, kindly provided by Dr. Larry Fisher, National Institute of Dental and Craniofacial Research, NIH, Bethesda, Md), Mac-2 (1:250, Cedarlane, Burlington, Canada), Ki-67 (1:50, Novus biological, Littleton, CO, USA), collagen neoepitope (1:100 IBEX Technologies, Montreal, Canada), biglycan (LF 159. 1:1000, kindly provided by Dr. Larry Fisher, National Institute of Dental and Craniofacial Research, NIH, Bethesda, Md), lumican (1:200, R&D Systems, Minneapolis, USA). The respective biotinylated secondary antibodies (1:1000) were obtained from Calbiochem. Detection was performed using diaminobenzidine (Zytomed, Berlin, Germany) as a chromogen. Nuclei were stained with hemalaun solution (Merck, Darmstadt, Germany).

Bright field images (8-bit) of one stained section per animal (each n representing one section of one animal) were captured using a Zeiss Axio Imager 2 at 100x magnification. Digital quantitative image analysis of the staining intensity was performed by a modified approach based on Dai *et al*.^52^ using ImageJ software 1.41 v (National Institutes of Health). The color channels of hemalaun and DAB was separated by the color deconvolution plug-in. Negative controls and strongly DAB-positive images were used to determine the thresholds and the analyzed range of signals in order to minimized the background interference and maximized the signal of DAB-positive tissue. Thresholds were kept constant for the complete analysis of one antigen. The measurement was performed in the papillary dermis excluding regions which contained hair follicles. Per skin section data from three randomly selected areas were averaged.

### Skin Parameters

Skin moisture and viscoelasticity (Ve) were measured before each biopsy using the DermaLab^®^ USB System (Cortex Techology, Denmark) according to the manufacturers protocol at constant temperature and atmospheric humidity.

### RNA Isolation and Quantification of Gene Expression

Total RNA was isolated using RNeasy total RNA kits (Qiagen,Hilden, Germany). The RNA concentration was determined via photometric measurement at 260/280. Aliquots of total RNA (1 μg) were applied for cDNA synthesis using SuperscriptIII first-strand synthesis system for reverse transcriptase-polymerase chain reaction (RT-PCR) (Invitrogen, Karlsruhe, Germany). In order to analyze the mRNA expression primers were designed employing Primer Express 3.0 software (Applied Biosystems, Darmstadt, Germany) based on published mRNA sequences. The sequences are given in Table 1. Each real time RT-PCR was performed in triplicates and the mean value calculated. PCR was carried out using SYBR Green PCR Master Mix (Applied Biosystems) as described [15]. The 2^[−ΔΔC(T)]^ method was used for comparison of the relative expressionin qRT-PCR between control and treated cells.

### Statistical Analysis

Statistical analyses were performed using MatLab R2015a (The MathWorks, Inc.) in combination with the Statistics and Machine Learning Toolbox™. In order to obtain normally distributed data, inverse hyperbolic sine transformation was performed. Next, a Generalized Linear Model (GLM) for 2 × 2 × 2 factorial treatment design was applied investigating all two- and three-level interactions. Tukey’s Honestly Significant Difference procedure was employed for pair-wise comparisons. For this study, a significance level of α = 0.05 was used.

## Additional Information

**How to cite this article**: Röck, K. *et al*. Chronic UVB-irradiation actuates perpetuated dermal matrix remodeling in female mice: Protective role of estrogen. *Sci. Rep.* **6**, 30482; doi: 10.1038/srep30482 (2016).

## Supplementary Material

Supplementary Information

## Figures and Tables

**Table 1:** Primer sequences.

Gene	Forward	Reverse
*Gapdh*	5′-TGGCAAAGTGGAGATTGTTGCC-3′	5′-AAGATGGTGATGGGCTTCCCG-3′
*Collagen1*	5′-CCTGGTAAAGATGGTGCC-3′	5′-CACCAGGTTCACCTTTCGCACC-3′
*Mmp3*	5′-TCAGTACCTTCCCAGGTTCG-3′	5′-GTCTTGGCAAATCCGGTGTA-3′
*Mmp9*	5′-CCTGAAAACCTCCAACCTCA-3′	5′-GCTTCTCTCCCATCATCTGG-3′
*Mmp13*	5′-GATGACCTGTCTGAGGAAGACC-3′	5′-CAAGAGTCGCAGGATGGTAGT-3′
*Has1*	5′-TATGCTACCAAGTATACCTCG-3′	5′-TCTCGGAAGTAAGATTTGGAC-3′
*Has2*	5′-CGGTCGTCTCAAATTCATCTG-3′	5′-ACAATGCATCTTGTTCAGCTC-3′
*Has3*	5′-GATGTCCAAATCCTCAACAAG-3′	5′-CCCACTAATACATTGCACAC-3′
*Vcan*	5′-ACCATGTCACTGGCTGTGG-3′	5′-AGCGGCAAAGTTCAGAGTGT-3′
*Bgn*	5′-CTGAGGGAACTTCACTTGGA-3′	5′-CAGATAGACAACCTGGAGGAG-3′
*Lum*	5′-TCGAGCTTGATCTCTCCTAT-3′	5′-TGGTCCCAGGATCTTACAGAA-3′

## References

[b1] Verdier-Sevrain S., Bonte F. & Gilchrest B. Biology of estrogens in skin: implications for skin aging. Exp Dermatol 15, 83–94 (2006). 16433679 10.1111/j.1600-0625.2005.00377.x

[b2] Fisher G. J. . Molecular basis of sun-induced premature skin ageing and retinoid antagonism. Nature 379, 335–339 (1996). 8552187 10.1038/379335a0

[b3] Archer D. F. Postmenopausal skin and estrogen. Gynecol Endocrinol 28 Suppl 2, 2–6 (2012). 22849791 10.3109/09513590.2012.705392

[b4] Thornton M. J. Estrogens and aging skin. Dermatoendocrinol 5, 264–270 (2013). 24194966 10.4161/derm.23872 PMC3772914

[b5] Bruls W. A., van Weelden H. & van der Leun J. C. Transmission of UV-radiation through human epidermal layers as a factor influencing the minimal erythema dose. Photochem Photobiol 39, 63–67 (1984). 6701210 10.1111/j.1751-1097.1984.tb03405.x

[b6] Quan T. . Matrix-degrading metalloproteinases in photoaging. J Investig Dermatol Symp Proc 14, 20–24 (2009). 10.1038/jidsymp.2009.8 PMC2909639 19675548

[b7] Verzijl N. . Age-related accumulation of Maillard reaction products in human articular cartilage collagen. Biochem J 350 Pt 2, 381–387 (2000). 10947951 PMC1221264

[b8] Varani J. . Inhibition of type I procollagen synthesis by damaged collagen in photoaged skin and by collagenase-degraded collagen *in vitro*. Am J Pathol 158, 931–942 (2001). 11238041 10.1016/S0002-9440(10)64040-0 PMC1850364

[b9] Sator P. G., Schmidt J. B., Rabe T. & Zouboulis C. C. Skin aging and sex hormones in women–clinical perspectives for intervention by hormone replacement therapy. Experimental dermatology 13 Suppl 4, 36–40 (2004). 15507111 10.1111/j.1600-0625.2004.00259.x

[b10] Koshiishi I., Horikoshi E. & Imanari T. Quantification of hyaluronan and chondroitin/dermatan sulfates in the tissue sections on glass slides. Analytical biochemistry 267, 222–226 (1999). 9918675 10.1006/abio.1998.3010

[b11] Stern R. & Maibach H. I. Hyaluronan in skin: aspects of aging and its pharmacologic modulation. Clinics in dermatology 26, 106–122 (2008). 18472055 10.1016/j.clindermatol.2007.09.013

[b12] Stern R. Complicated hyaluronan patterns in skin: enlightenment by UVB? J Invest Dermatol 127, 512–513 (2007). 17299433 10.1038/sj.jid.5700605

[b13] Weigel P. H., Hascall V. C. & Tammi M. Hyaluronan synthases. J Biol Chem 272, 13997–14000 (1997). 9206724 10.1074/jbc.272.22.13997

[b14] Tammi R., Pasonen-Seppanen S., Kolehmainen E. & Tammi M. Hyaluronan synthase induction and hyaluronan accumulation in mouse epidermis following skin injury. J Invest Dermatol 124, 898–905 (2005). 15854028 10.1111/j.0022-202X.2005.23697.x

[b15] Sudel K. M. . Tight control of matrix metalloproteinase-1 activity in human skin. Photochemistry and photobiology 78, 355–360 (2003). 14626663 10.1562/0031-8655(2003)078<0355:tcomma>2.0.co;2

[b16] Dai G. . Chronic ultraviolet B irradiation causes loss of hyaluronic acid from mouse dermis because of down-regulation of hyaluronic acid synthases. Am J Pathol 171, 1451–1461 (2007). 17982124 10.2353/ajpath.2007.070136 PMC2043507

[b17] Takahashi M., Funahashi T., Shimomura I., Miyaoka K. & Matsuzawa Y. Plasma leptin levels and body fat distribution. Horm Metab Res 28, 751–752 (1996). 9013757 10.1055/s-2007-979893

[b18] Evanko S. P., Angello J. C. & Wight T. N. Formation of hyaluronan- and versican-rich pericellular matrix is required for proliferation and migration of vascular smooth muscle cells. Arteriosclerosis, thrombosis, and vascular biology 19, 1004–1013 (1999). 10.1161/01.atv.19.4.1004 10195929

[b19] Knott A. . Deregulation of versican and elastin binding protein in solar elastosis. Biogerontology 10, 181–190 (2009). 18704747 10.1007/s10522-008-9165-3

[b20] Bernstein E. F. . Differential expression of the versican and decorin genes in photoaged and sun-protected skin. Comparison by immunohistochemical and northern analyses. Lab Invest 72, 662–669 (1995). 7783424

[b21] Iozzo R. V. Matrix proteoglycans: from molecular design to cellular function. Annual review of biochemistry 67, 609–652 (1998). 10.1146/annurev.biochem.67.1.609 9759499

[b22] Kao W. W., Funderburgh J. L., Xia Y., Liu C. Y. & Conrad G. W. Focus on molecules: lumican. Experimental eye research 82, 3–4 (2006). 16213485 10.1016/j.exer.2005.08.012 PMC2876311

[b23] Nastase M. V., Young M. F. & Schaefer L. Biglycan: a multivalent proteoglycan providing structure and signals. The journal of histochemistry and cytochemistry: official journal of the Histochemistry Society 60, 963–975 (2012). 22821552 10.1369/0022155412456380 PMC3527886

[b24] Podskochy A., Koulikovska M., Fagerholm P. & van der Ploeg I. Biglycan gene expression in UVR-exposed rabbit corneas. Acta Ophthalmol Scand 82, 200–204 (2004). 15043541 10.1111/j.1600-0420.2004.00232.x

[b25] Brincat M. . Skin collagen changes in postmenopausal women receiving different regimens of estrogen therapy. Obstetrics and gynecology 70, 123–127 (1987). 3601260

[b26] Castelo-Branco C., Duran M. & Gonzalez-Merlo J. Skin collagen changes related to age and hormone replacement therapy. Maturitas 15, 113–119 (1992). 1345134 10.1016/0378-5122(92)90245-y

[b27] Son E. D. . Topical application of 17beta-estradiol increases extracellular matrix protein synthesis by stimulating tgf-Beta signaling in aged human skin *in vivo*. J Invest Dermatol 124, 1149–1161 (2005). 15955089 10.1111/j.0022-202X.2005.23736.x

[b28] Rock K. . Estradiol protects dermal hyaluronan/versican matrix during photoaging by release of epidermal growth factor from keratinocytes. J Biol Chem 287, 20056–20069 (2012). 22493503 10.1074/jbc.M112.353151 PMC3370189

[b29] Fisher G. J. . Pathophysiology of premature skin aging induced by ultraviolet light. The New England journal of medicine 337, 1419–1428 (1997). 9358139 10.1056/NEJM199711133372003

[b30] Lee Y. J. . Effect of estrogen on the expression of matrix metalloproteinase (MMP)-1, MMP-3, and MMP-13 and tissue inhibitor of metalloproternase-1 in osteoarthritis chondrocytes. Rheumatology international 23, 282–288 (2003). 12684836 10.1007/s00296-003-0312-5

[b31] Rittie L. & Fisher G. J. UV-light-induced signal cascades and skin aging. Ageing Res Rev 1, 705–720 (2002). 12208239 10.1016/s1568-1637(02)00024-7

[b32] Vogel K. G. & Trotter J. A. The effect of proteoglycans on the morphology of collagen fibrils formed *in vitro*. Coll Relat Res 7, 105–114 (1987). 3621881 10.1016/s0174-173x(87)80002-x

[b33] Geng Y., McQuillan D. & Roughley P. J. SLRP interaction can protect collagen fibrils from cleavage by collagenases. Matrix Biol 25, 484–491 (2006). 16979885 10.1016/j.matbio.2006.08.259

[b34] Corsi A. . Phenotypic effects of biglycan deficiency are linked to collagen fibril abnormalities, are synergized by decorin deficiency, and mimic Ehlers-Danlos-like changes in bone and other connective tissues. J Bone Miner Res 17, 1180–1189 (2002). 12102052 10.1359/jbmr.2002.17.7.1180

[b35] Brenneisen P., Wenk J., Wlaschek M., Krieg T. & Scharffetter-Kochanek K. Activation of p70 ribosomal protein S6 kinase is an essential step in the DNA damage-dependent signaling pathway responsible for the ultraviolet B-mediated increase in interstitial collagenase (MMP-1) and stromelysin-1 (MMP-3) protein levels in human dermal fibroblasts. J Biol Chem 275, 4336–4344 (2000). 10660603 10.1074/jbc.275.6.4336

[b36] Varani J. . Vitamin A antagonizes decreased cell growth and elevated collagen-degrading matrix metalloproteinases and stimulates collagen accumulation in naturally aged human skin. J Invest Dermatol 114, 480–486 (2000). 10692106 10.1046/j.1523-1747.2000.00902.x

[b37] Lee Y. R. . Cordycepin inhibits UVB-induced matrix metalloproteinase expression by suppressing the NF-kappaB pathway in human dermal fibroblasts. Exp Mol Med 41, 548–554 (2009). 19381070 10.3858/emm.2009.41.8.060 PMC2739894

[b38] Verdier-Sevrain S., Bonte F. & Gilchrest B. Biology of estrogens in skin: implications for skin aging. Exp Dermatol 15, 83–94 (2006). 16433679 10.1111/j.1600-0625.2005.00377.x

[b39] Sator P. G. . A prospective, randomized, double-blind, placebo-controlled study on the influence of a hormone replacement therapy on skin aging in postmenopausal women. Climacteric: the journal of the International Menopause Society 10, 320–334 (2007). 17653959 10.1080/13697130701444073

[b40] Kanda N. & Watanabe S. Regulatory roles of sex hormones in cutaneous biology and immunology. Journal of dermatological science 38, 1–7 (2005). 15795118 10.1016/j.jdermsci.2004.10.011

[b41] Tsukahara K. . Ovariectomy is sufficient to accelerate spontaneous skin ageing and to stimulate ultraviolet irradiation-induced photoageing of murine skin. The British journal of dermatology 151, 984–994 (2004). 15541076 10.1111/j.1365-2133.2004.06203.x

[b42] Bourguignon L. Y., Gilad E., Rothman K. & Peyrollier K. Hyaluronan-CD44 interaction with IQGAP1 promotes Cdc42 and ERK signaling, leading to actin binding, Elk-1/estrogen receptor transcriptional activation, and ovarian cancer progression. J Biol Chem 280, 11961–11972 (2005). 15655247 10.1074/jbc.M411985200

[b43] Kawashima H. . Binding of a large chondroitin sulfate/dermatan sulfate proteoglycan, versican, to L-selectin, P-selectin, and CD44. J Biol Chem 275, 35448–35456 (2000). 10950950 10.1074/jbc.M003387200

[b44] Wu Y. . Versican protects cells from oxidative stress-induced apoptosis. Matrix Biol 24, 3–13 (2005). 15748997 10.1016/j.matbio.2004.11.007

[b45] Halliday G. M. Inflammation, gene mutation and photoimmunosuppression in response to UVR-induced oxidative damage contributes to photocarcinogenesis. Mutat Res 571, 107–120 (2005). 15748642 10.1016/j.mrfmmm.2004.09.013

[b46] Stern R., Kogan G., Jedrzejas M. J. & Soltes L. The many ways to cleave hyaluronan. Biotechnol Adv 25, 537–557 (2007). 17716848 10.1016/j.biotechadv.2007.07.001

[b47] Vij N., Roberts L., Joyce S. & Chakravarti S. Lumican regulates corneal inflammatory responses by modulating Fas-Fas ligand signaling. Invest Ophthalmol Vis Sci 46, 88–95 (2005). 15623759 10.1167/iovs.04-0833

[b48] Longaker M. T. . Studies in fetal wound healing. V. A prolonged presence of hyaluronic acid characterizes fetal wound fluid. Ann Surg 213, 292–296 (1991). 2009010 10.1097/00000658-199104000-00003 PMC1358347

[b49] Ashcroft G. S., Horan M. A. & Ferguson M. W. Aging is associated with reduced deposition of specific extracellular matrix components, an upregulation of angiogenesis, and an altered inflammatory response in a murine incisional wound healing model. J Invest Dermatol 108, 430–437 (1997). 9077470 10.1111/1523-1747.ep12289705

[b50] Stevenson S., Nelson L. D., Sharpe D. T. & Thornton M. J. 17beta-estradiol regulates the secretion of TGF-beta by cultured human dermal fibroblasts. J Biomater Sci Polym Ed 19, 1097–1109 (2008). 18644234 10.1163/156856208784909354

[b51] Freudenberger T. . Proatherogenic effects of estradiol in a model of accelerated atherosclerosis in ovariectomized ApoE-deficient mice. Basic Res Cardiol (2010). 10.1007/s00395-010-0091-6 20177692

[b52] Dai G. . Chronic ultraviolet B irradiation causes loss of hyaluronic acid from mouse dermis because of down-regulation of hyaluronic acid synthases. The American journal of pathology 171, 1451–1461 (2007). 17982124 10.2353/ajpath.2007.070136 PMC2043507

[b53] Danielson K. G. . Targeted disruption of decorin leads to abnormal collagen fibril morphology and skin fragility. J Cell Biol 136, 729–743 (1997). 9024701 10.1083/jcb.136.3.729 PMC2134287

[b54] Yaar M. & Gilchrest B. A. Photoageing: mechanism, prevention and therapy. The British journal of dermatology 157, 874–887 (2007). 17711532 10.1111/j.1365-2133.2007.08108.x

