# Supplementary figures and images for: Chronic UVB-irradiation actuates perpetuated dermal matrix remodeling in female mice: Protective role of estrogen
Source: Sci Rep. 2016 Jul 27;6:30482. doi: 10.1038/srep30482 (PMC4962040; doi:10.1038/srep30482)

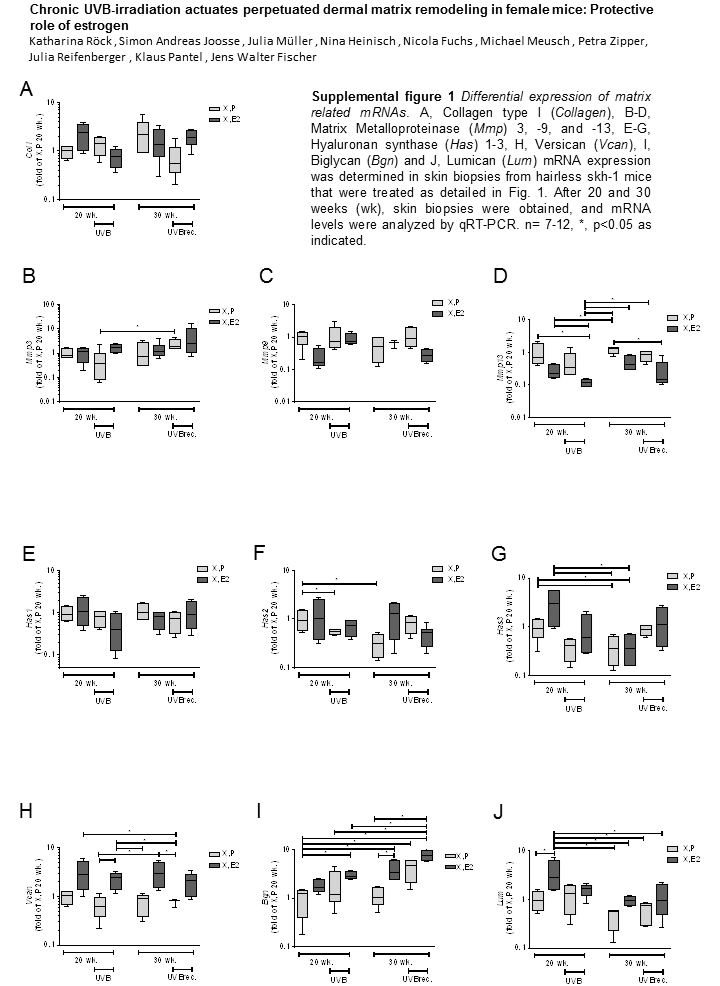

Supplement: Supplementary Information [file srep30482-s1.tiff]
